# Psychosis risk research versus daily prognosis uncertainties: A qualitative study of French youth psychiatrists’ attitudes toward predictive practices

**DOI:** 10.1371/journal.pone.0179849

**Published:** 2017-07-19

**Authors:** Laelia Benoit, Marie Rose Moro, Bruno Falissard, Nicolas Henckes

**Affiliations:** 1 Department of Psychiatry at Cochin hospital, Paris, France; 2 CESP, INSERM U1018, Université Paris-Saclay, Univ. Paris-Sud, UVSQ, Univ. Paris-Descartes, Paris, France; 3 CERMES3, CNRS, Villejuif, France; Maastricht University, NETHERLANDS

## Abstract

**Background:**

Over the last twenty years, predicting psychosis has become a priority of both research and policies. Those approaches include the use of the At Risk Mental State category (ARMS) and of standardized predictive tools. In comparison to most developed countries, early interventions programs are only little developed in France. However, cases of young patients presenting unclear symptoms that might be a beginning psychosis or might as well reflect some adolescent unease are commonplace in psychiatry. Yet little is known about the routine practices of youth psychiatrists regarding psychosis risk management. Do they anticipate mental disorders?

**Method:**

The Grounded Theory is an agreed-upon qualitative method in social science field that links subjective experiences (individual narratives) to social processes (professional norms and mental health policies). 12 French youth psychiatrists were interviewed about psychosis early management and their daily prognosis practices with teenagers.

**Results:**

If all participants were aware of early intervention programs, most of them did not make use of standardized scales. Psychiatrists’ reluctance toward a psychosis risk standardized assessment was shaped by three difficulties: first the gap between theoretical knowledge and practice; second their impossibility to make reliable prognoses; and third, the many uncertainties surrounding medical judgment, adolescence and the nature of psychosis. Nevertheless, they provided their young patients with multiple months follow up without disclosing any risk category.

**Conclusion:**

Anticipating a psychosis onset remains a highly uncertain task for psychiatrists. In France, psychiatrists’ inconspicuous risk management might be supported by the universal costs coverage that is not conditional on a diagnosis disclosure.

## Introduction

Over the last twenty years the rise of Early Intervention in Psychiatry has stimulated the creation of centers specialized in the assessment and treatment of patients at an early stage or even at risk of psychosis [1–3]. Yet in many countries these centers are not accessible to all patients, who, as a consequence, are seen by psychiatrists who do not propose a standardized assessment of the risk condition. Even when a specialized consultation is accessible, a psychiatrist might have to decide whether the unclear symptoms presented by his patient might be the early signs of a psychosis or not in order to refer him for assessment. Little is known, however, regarding the attitudes of psychiatrists towards these situations.

This study was conducted in France where centers of expertise in early interventions are not fully developed. If there has been an interest from the media and the higher health administration in early intervention strategies [4–6], today only a few centers offer assessment and care for prodromal patients, most of them in the Paris region [7]. On another hand youth psychiatry is well developed thanks to the existence of an array of specialized facilities, including university consultations centers, adolescent health centers combining different specialized consultations—such as psychiatry, general medicine gynecology, dermatology -, as well as psychiatric consultations in many community mental health centers and private consultation. France has among the highest number of practicing psychiatrists and a strong influence of psychoanalysis. Universal coverage of health costs creates a system accessible to young people. In order to grasp the present implementation of early interventions, a local assessment of daily practices appears particularly relevant.

According to Christakis’ seminal work on prognosis in medicine, two different concepts of “prognosis” can be distinguished [8]. On the one hand “prognosis” refers to the theoretical course of a given disorder, as it is described for instance in handbooks. In that sense, even if there is scientific evidence that its course is more favourable than traditionally thought [9–12], schizophrenia remains usually conceived as a chronic disease in the public opinion; while depression, for instance, should not last longer than a few months. A second meaning of prognosis refers to the practice of envisioning the future course of a disorder in a given patient. In this meaning prognosis might be broken down into three components:

- the first is foreseeing, that is envisioning the future of this particular patient;
- the second component consists of anticipating, that is acting on the basis of this prevision in order to prevent or manage an event that has to come;
- the last component of prognosis is communicating to the patient this prevision, in other words “predicting”.

In this second meaning, prognosis is a major dimension of medical practice, and yet it remains understudied. In a sociological perspective, this interdisciplinary study does not aim at providing a review of scientific evidence of early intervention efficiency. We evaluated a sample of French adolescent psychiatrists’ contemporary views regarding early intervention. We focused on their daily practice of prognosis: To what extent do they think that psychosis may be anticipated? How do they themselves make anticipations about the future of their patients?

## Methods

Participants were contacted via an email sent to the team mailing list of 4 public psychiatry departments of the Paris Region and of 2 community mental health centers. The same email was sent to two private practice contact-list to patients in the public services and the mailing list of a professional society (French Society of Child and Adolescent Psychiatry, SFPEADA). The email was titled “Anticipating young patients’ future: is prognosis part of your practice?” and proposed a one hour long interview on questions of prognosis. No retribution was offered. Twelve youth psychiatrists (6 women, 6 men) aged from 28 to 70, and working in a variety of settings agreed to participate. In order to ensure confidentiality, numbers were used in this article to refer to participants, Table 1. The interviews took place at their office and were semi-structured, using a schedule comprising a series of themes to be addressed during the session. The schedule was developed after a review of the relevant psychiatric and sociological literature. The questions were meant to focus on the problem of psychosis risk and open a free dialogue about its daily management: when you see a teenager presenting unclear symptoms that may be early signs of psychosis, do you feel able to predict psychotic transition? If not, do you however expect some difficulties? What appears a priority for his future? Do you make use of prevalence statistics or specific tools to assess psychosis risk? Do you refer your patients to expert centers? The interviews were recorded, fully transcribed and analyzed using the Grounded Theory (GT) methodology [13]. Inspired by ethnographical studies then theorized by Glaser and Strauss [14], GT is an agreed-upon standard for social sciences research since the 1960’s [15]. The Grounded Theory links social structures with processes occurring at an individual level by focusing on themes that represent underlying phenomena, interactions and their consequences [13]. As in other inductive methods, it is not necessary to define an exact number of respondents before the research begins. The data are coded in order to generate categories which are then validated through constant comparisons as new interviews are done [16]. Thus data analysis, further sampling and theoretical development are simultaneously developed until saturation is reached allowing to build a new theory about the subject of research [14]. To ensure reliability, the analysis is repeated by more than one researcher, a process which is called triangulation [17]. In this study, two researchers (LB and NH) independently coded and analyzed all data [18], and their findings were discussed during meetings of our research team (LB, NH, MRM, BF).

**Table 1:** Participants.

Interview number	Sex	Age	Setting(s): Paris Region	Drug prescription	Aware of early intervention programs and center of expertise	Interest in early intervention research	Already worked in partnership with early detection centers	Involvement in research: personal involvement or partnership with a research team in youth psychiatry
**1**	F	55	Public youth psychiatry consultation center Private practice psychoanalysis	No	Yes	No	No	No
**2**	M	60	Public consultation center for children	Yes	Yes	No	No	Pharmacology history
**3**	M	35	Student help Public youth psychiatry consultation center	No	Yes	No	No	No
**4**	M	70	Public youth psychiatry consultation center Private practice, psychoanalysis	No	Yes	No	No	No
**5**	M	70	Public youth psychiatry consultation center	Yes	Yes	Yes	No	No
**6**	F	50	Public center of expertise for language disorder. Private practice of psychoanalysis	No	Yes	Yes	Yes	Cohort of children with dyspraxia in order to assess psychosis onset rates in adolescence and young adulthood.
**7**	F	42	Public youth psychiatry department of a University Hospital Center.	Yes	Yes	Yes	Yes	Cohort of teens after a first bipolar episode or psychotic episode
**8**	M	32	Public youth psychiatry department of University Hospital Center.	Yes	Yes	No	Yes	Qualitative studies of suicide among teens
**9**	F	50	Public youth psychiatry consultation center	Yes	Yes	Yes	Yes	Qualitative studies about ADHD
**10**	M	40	School-based residential psychiatry unit	Yes	Yes	No	Yes	No
**11**	F	50	School-based residential psychiatry unit	Yes	Yes	Yes	Yes	No
**12**	F	28	Public youth psychiatry consultation center.	Yes	Yes	No	No	Pharmacological management strategies for resistant psychosis.

## Results

As part of our sociological perspective, this results section reflects the opinions of the interviewees—not the authors’ ones. For instance, we asked questions related to psychosis, but participants answered focusing on schizophrenia. Later on, the discussion section will analyze these views in the light of both medical sociology and the field of ARMS research.

### The gap between research and practice

If all participants were aware of early intervention programs, most of them did not make use of predictive scales such as the CAARMS or the SIPS [19], nor did they refer their patients to early management centers. Actually, it would not make sense for all practitioners to use them, as these scales require extensive training. While ARMS assessment instruments do not predict psychosis in an individual, our participants think that they have the ambition to do so, and criticize it. Thus, they are reluctant to refer their patients to early management centers where ARMS might be assessed. However, either trained or not, all participants provided their young patients with multiple months follow up without use of an At Risk Mental State category (ARMS) [20,21].

#### 1. Scales do not bring certitude

Participants were either enthusiastic about ARMS research or not. Half of them had already worked in partnership with an early detection center, for research or clinical purposes [Table 1]. Nevertheless, most psychiatrists did not use standardized tools to assess prodromal conditions.

« I know the center of expertise. But I prefer to handle things myself a while, even with the first months fuzziness, than to expose the patient and his family to a check-list concluding “you are psychotic”. Well, even colleagues working in those centers say that one should be careful, that there is no 100% guarantee. »

(psychiatrist number 5—see Table 1)

Only one psychiatrist used the Comprehensive Assessment of At Risk Mental States (CAARMS) among various diagnostic instruments in her daily work. Nevertheless, she stressed that assessment results did not bring her any certitude about the problems a young patient experienced.

“I followed this patient for 4 years, until his late teens. In the end, I am still not sure about his diagnosis. Parents reclaim standardized assessments. So we assess, we check things up, we give a sort of diagnostic listing! [laugh] But I am still not sure. Well, anyway, we evaluated.»

(7)

#### 2. Statistics do not help to predict psychosis

Even psychiatrists involved in outcome studies acknowledged a difficulty to match both statistical data and clinical sense.

« For this girl, I would say that it will turn into a bipolar disorder. But we conducted a follow up study among 80 teens diagnosed with a bipolar disorder type I. As adults, a *third* of them had an onset of psychosis! […] So, it brings a self-dialectic process, because I really feel that it will turn into a bipolar disorder”.

(7)

A psychiatrist explained that outcome results from early intervention research must not be disclosed to parents.

« I know that kids with dyspraxia awake researchers’ interest. But I just can’t say to parents: ‘‘research teams believe that there is 30% risk of psychosis onset during adolescence”! We may think it, we may conduct studies about it, but in daily work this is not possible. Anyhow, I keep an eye on them, I follow them up. »

(6)

### Impossible to predict psychosis in daily work

#### Detection: An inescapable question without an answer

All psychiatrists considered prognosis as an unavoidable question but they stressed the difficulty to remain informed of their young patients’ outcomes.

« I would say that prognosis is always a question, but we have no answer. We receive no news, but is it because the young people recovered or is it because they are somewhere else?”

(10)

Some psychiatrists preferred to avoid this question but fail to do so.

« It isn’t useful for me to imagine that a 15 years old teen may become schizophrenic. It is pointless.

- Ok. But do you still wonder about his future or not?

- Yes! Yes. »

(2)

#### Prevention: no proof of success

Interviewees expressed doubts that early intervention could really prevent the onset of psychosis.

«—*Is it possible to prevent psychosis onset*?

- (Laughing) Good question! I would be curious to hear other psychiatrists’ answers. (Serious again) I don’t know. I don’t know. I don’t know. It would be hard to prove. »

(8)

Most psychiatrists actually consider that early intervention should be seen as a form of tertiary prevention (aiming at reducing permanent impairment) rather than a strategy of primary or secondary prevention (avoiding the onset of psychosis) [22]. Despite increasing waiting lists, the French mental health system has been designed to provide free and accessible primary care since 1960 [23]. In this context, it appears unethical to evaluate the efficacy of early intervention on patients with subdued symptoms through a comparative outcome study.

« *Can psychiatric care avert psychosis onset*?

- What a question! I don’t know. One can assume that without proper care—I don’t know if they would really develop schizophrenia—but they would surely have a psychological or cognitive impairment. Without care at all, I will never know. It would mean venturing a study following-up persons who receive care, and the same number who not receive any care, and assess the natural course. That is questionable, ethically. »

(10)

#### No theory spares worries about the future

Some participants spontaneously explained their reluctance toward predictive approaches by a theoretical choice such as psychoanalysis or a adolescent psychiatrist’s view. Nevertheless, this attitude did not spare them worries about the future of their young patients, and failed to provide an unequivocal answer to the prognostic question.

« As a psychotherapist I should break free from [the prognosis] question to elaborate the sense of what is happening to him. But having in mind that he might be somehow delirious […] makes me suspicious of what may happen. I referred him to a colleague for a prescription so as to break free from this problem. But, of course, I was not relieved! »

(1)

« The adolescent psychiatrist attitude is to face teenagers’ immediate shifts. It is different from the attitude of an adult psychiatrist. [The long-term risk] is obviously a question when we stop and think about it, but not in our daily work. It is important to be far-sighted, but it is difficult.»

(8).

### Three sources of uncertainty

Psychiatrists’ impossibility to predict psychosis emerges from three sources of uncertainty.

#### Medical judgment

Each case is an individual one: Outcome statistics do not allow reliable predictions for a given individual. Psychiatrists’ uncertainty emerges from the limits of their own knowledge, the limits of medical science, and their difficulty to distinguish between both sources of uncertainty.

«[*Speaking about outcome after a first psychotic episode*] There is one third, one third, one third *[in reference to 3 possible outcomes*: *psychosis*, *recurrent acute psychotic episodes*, *and no mental trouble]*. But—unless you know new studies—there is no clue to guess in which third is your patient”.

(8)

Feeling relieved by uncertainty: Uncertainty disclosure may be used as a way to manage teenagers and parents expectancies.

“I tell parents that I am not a soothsayer! There are three outcomes, but there is no way to know how it will evolve”

(8)

Uncertainty also supports psychiatrists’ curiosity toward their patients’ evolution, and relieves them from the burdening responsibility of an unreliable prophecy.

“If you tell me what the magic formula is to say “this one will be psychotic”, I would be glad to have it! But it would also make the job rather boring. That is something interesting in our job. Not to know. »

(8)

#### Adolescence process

Is it teenage rebellion or mental trouble? Psychiatrists acknowledge having difficulties to distinguish what may reflect adolescent unease and what may be a true psychiatric disorder.

“It is hard to set thing apart, from what is teenage rebellion and what is a deep psychic crisis. We are always providing a counterweight on the one side or on the other side.”

(10)

The adolescence processappears in between instability and self-construction.Youth psychiatrists express the feeling that teenagers are moving on an irretrievable trajectory that can be improved through psychiatric support.

« One gets structured, during adolescence and young adulthood by symptoms and mental suffering. When you work with teens you have this feeling that your influence allows them not to switch on the wrong side”.

(4)

Nevertheless, daily practice consists in dealing with immediate changes that make every short or long term outcome unpredictable.

« When you think of something, the teen has already changed! »

(8)

#### Psychosis uncertainties

A historical vision of psychosis as a lifelong mental and social trouble shapes psychiatrists reluctance to anticipate psychotic transition.

«One may be afraid to anticipate an illness that you do not still have. It is a serious problem, severe, resistant, with heavy treatments, side effects for sure, that impacts social and professional life. It will be a lifelong problem, and he is only a teen!”

(12)

Psychiatrists face a paradox. The success of early intervention would contradict the vision of this mental trouble as a social impairment.

« Persons that turn out « normal » again a few years later, I am forced to consider that I was mistaken about a schizophrenia early diagnosis. »

(5)

Both eventualities of false positive patients and cases of successful care lead psychiatrists to avoid prediction and refuse to disclose an ARMS status.

“How many teens have been labeled schizophrenics? Time goes by and reveals that they aren’t schizophrenics. Either it wasn’t schizophrenia or they managed to recover. That is harmful. If we have a doubt it is better not to mention it.”

(10)

Thus, the uncertainties surrounding medical practice, adolescence and psychosis shape youth psychiatrists’ impossibility to predict psychosis.

## Discussion

According to participants, neither the results of research, standardized instruments nor clinical judgment enable the prediction of psychosis before its onset. The gap between ARMS research and daily work is related to the limitations of preventive instruments, as well as that of schools of thought. For a given teenager, detection and prevention efficiency remain impossible to prove. Nevertheless, neither psychoanalysis nor child development theories—both focusing on a present timeframe in order to support teenagers’ elaboration and positive identity construction [24]–spare psychiatrists worries about their young patients future. Finally, individual psychosis prediction failure appears related to the many uncertainties surrounding medical practice, the adolescence process, and psychosis itself. In the following sections, we propose to discuss participants’ uncertainties through two ideas. First, individual prognosis is a challenge inherent to medical practice. Second, declared interest for or reluctance toward predictive approaches may be shaped by health systems.

### The challenge of individual psychosis prediction

The first source of uncertainty mentioned by participants refers to medical judgment and is consistent with the sociological analysis of medicine. Fox argued that learning to cope with the uncertainty of medical practice is a professional norm that is assimilated early in the curriculum. She distinguished three sources of uncertainty: the limits of a physician’s knowledge, the limits of medical science, and the inability of the physician to distinguish between those two sources of uncertainty [25,26]. Freidson and Hunter argued that medicine is taught as a science of individuals. Each clinical case is unique and therefore uncertain [27,28]. According to Light, medical trainees learn to control uncertainty through specialization, involvement in schools of thought, clinical experience, and growing autonomy [29]. Thus, claiming to belong to a school of thought such as psychoanalysis or youth psychiatry could merely be way to cope with uncertainty. Dealing with serious diseases, prognosis uncertainty can be functional, that is used by doctors in order to avoid distress reactions from patients and their relatives [30] and avoid being held responsible for the patient’s outcome [8]. In that sense, a prognosis disclosure is a too risky commitment. This opinion is expected to persist over some period of time, to account for the psychiatrist’s “consistent behavior”. According to Becker, commitments come into being when a person, by making a side bet, links extraneous interests with a consistent line of activity [31]. Side bets are often a consequence of the person’s participation in social organizations. In daily work, a person sometimes finds that he has made side bets constraining his present activity because the existence of general cultural expectations provides penalties for those who violate them [31]. For instance, the cultural expectation about the behavior accounting for a practitioner’s trustworthiness is different from the expectation accounting for the trustworthiness of a researcher. People feel that a practitioner ought not to change his opinion too often and that one who does is inexperienced and untrustworthy [8]. On the contrary, a researcher’s shifting views on a subject might account for his commitment through his consistency in improving his study design. Thus, a practitioner will avoid prognosis disclosure, instead of sharing his worries about a psychosis onset to a young patient and then changing his opinion after a second appointment. Functional uncertainty is thus used by doctors in order to diminish their own level of commitment.

The second source of uncertainty mentioned by the participants to this study was the immediacy and unpredictability of teenagers’ symptoms. The adolescent period is seen as a social, hormonal, neurological, and psychological storm both by public opinion and by health professionals [32,33]. As early as in 1958, Anna Freud famously suggested that calm teenagers may hide distressful emotions: “being normal during the adolescence period is abnormal” [34]. The participants in our study argued that they were not always able to distinguish adolescent unease or rebellion from subdued psychotic symptoms. Interviewees’ vision of adolescence as an unpredictable process combining daily mood instability and social skills development is supported by neurosciences [35,36]. Teenagers new access to abstract thinking, if formerly stressed by child development psychology [37] is now supported by brain imagery studies revealing a neuronal network reconfiguration through synaptic trimming [38] that extends into the second and third decades of life [39]. Another difficulty is related to the fact that the incidence of positive psychotic experiences in the general population is 100 times greater than the incidence of psychotic disorders [40]. Moreover, in the adolescent and young adult population, psychotic-like experiences are strongly correlated with distress, depression and poor functioning [41].

Participants’ third source of uncertainty was related to psychosis diagnosis and course. First, we asked questions focusing on psychosis but participants’ answers focus on schizophrenia (in French “schizophrénie”) instead of psychosis (psychose). According to the Grounded Theory, this is neither a methodological problem, nor a translation mistake, but a meaningful result. The use of schizophrenia as a synonym for psychosis enlightens participants’ contemporary representation of psychosis as an equivalent of schizophrenia. The schizophrenia label, if still used as a reliable research category [42,43] is increasingly contested in order to reduce stigma [44] and account for a complex and poorly understood etiology [45,46]. The Ultra High Risk of psychosis (UHR) low prediction accuracy (15% to 25%) [47], high rates of false positive identifications (from 50% to 84%) [48,49] and difficulties to evaluate if early treatments improve long-term outcomes are stressed as current research challenges [50–54]. Research teams believe that the risk of psychosis onset during adolescence is far lower than 30% when SIPS or CARMS is applied in the general community [49]. Authors attribute this low predictive value to low prevalence of the psychosis risk syndrome in a general adolescent clinical population [55]. Thus, given the potential stigma associated to a Psychosis Risk syndrome [56] and its need to be further validated, it was not included in the DSM-V [57]. Second, participants expressed a contradiction between the practice of a probabilistic judgment in early diagnosis, due to the fact that psychosis diagnosis is based on a retrospective duration criteria [58]. Third, our results show that the vision of psychosis as a disastrous life-long trouble [59,60] refrains psychiatrists’ involvement in preventive approaches. Despite activism by former patients and patient advocates who have argued that psychosis did not prevent persons to live meaningful lives and to maintain a social role in their community, as well as scientific evidences that the course of schizophrenia is not always negative [9–12,61,62], schizophrenia’s major economic costs and duration shapes the idea that “*the onset of psychotic illness represents potential personal disaster in the life-course of an individual*” [63]. Thus, interviewed psychiatrists extended the deadline before disclosing such a dreadful perspective. However, if the primary outcome of interest in at-risk research was the development of psychotic disorder—whereby patients are categorized as either having ‘transitioned’ or ‘not transitioned’–recent research suggest that ‘non transitioned’ persons may have a poorer quality of life [64,65]. More than attenuated psychotic symptoms (APS), social and functioning difficulties as well as depressive symptoms can be the highest sources of distress leading UHR patients to seek help [66]. Thus, the term ‘outcome’ might be broadened to incorporate non-psychotic diagnoses, functioning and negative symptoms [67].

### Health systems beyond the research/clinic dichotomy

Interactionism in sociology focuses on the way that people act and on the reasons they give to justify their action [68]. In this perspective, a meaning does not need to be “true” to be effective. The meaning that people give to a thing or an event determines its symbolic reality, which has the power to induce a social effect.

Which reasons can be used by our participants to support their views? Even if they know statistical data related to psychosis onset, in the end psychiatrists make decisions according to their clinical sense. This “in-between” strategy including both rational and non-rational strategies has been proven to be efficient in risk-management strategies [69]. Our results show that participants do not expect that early diagnosis tools improve the actual care provided. They legitimize their reluctance toward ARMS assessment by the clinic/research dichotomy. For instance, two reasons are used by our participants to avoid the referral of their young patients to assessment centers: first, it is impossible to predict an individual outcome; second, it is impossible to know if psychosis can be prevented in an individual. However, researchers of the ARMS field already agree with those critics [49]: first, ARMS assessment instruments aim at determining an ARMS, not predicting psychosis in an individual. Second, early interventions for ARMS individuals aim at improving functional outcome at group level. Nevertheless, “valid” or not in a scientific meaning, these reasons induce a real social effect: our participants’ reluctance to refer their young patients for ARMS standardized assessment. According to the Grounded Theory, this confusion does not reveal a methodological mistake or participants’ lack of perspective, but a meaningful result. Moreover, we will argue in the following section that participants’ inconspicuous risk management might as well be understood considering the characteristics of the French health system.

How do our participants actually act? When they suspect a psychosis onset, interviewees start an inconspicuous risk management: multiple months follow up of this fuzzy clinical state. We suggest that this strategy may be influenced by the organization of youth mental health services in France. In this country 77% of mental health care is provided by the public system with a universal costs coverage [70]. A diagnosis of risk is not necessary in order to obtain reimbursement of mental health costs. Thus, psychiatrists use their subjective judgment to start a discreet follow-up. As early as in 1941, Rümke described the “praecox feeling” as a feeling of unease emanating in the interviewer that reflects the detachment of the patient and the failure of an ‘affective exchange’ [71]. Praecox feeling reliability has mainly been evaluated among persons with an ongoing psychosis. Compared with the standardized diagnostic classification the precision of the intuitive reasoning appears remarkably high [72–74]. On the contrary, in a U.H.R. population, Nelson and Yung showed that the *clinical impression* of an incipient psychosis—that is, a clinical judgment that a particular patient may soon progress to a full-threshold disorder—is not sufficient for predicting a psychosis outcome and may lead physicians to improper antipsychotic medication [75]. This result stresses the need to design more adequate follow ups for young people. Youth psychiatry (child and adolescent) services usually stop following patients when they reach 18 years of age and are then referred to adult services. The communication between those services remains limited both for clinical and for research purposes. Today, despite psychosis peak onset in late adolescence—early adulthood [76], only few youth psychiatry departments are designed for following up young patients until 25 to 30 years. The participants to our study usually did not receive news from their patients after they had been referred. The lack of outcome studies following young patients from child to adult departments and of services specially designed for persons aged 15–30, increases the youth psychiatrists’ challenge to imagine young patients’ evolution after their 18s.

### Study limitations and implications: Rationale for a comparison of local mental health system changes

If qualitative methods’ validity is increasingly acknowledged in medicine, the Grounded Theory (GT) remains rarely known in the medical field. If Interpretative Phenomenological Analysis (IPA) is the dominant method increasingly used in psychological studies—and previously employed by our team [77]–its’ main difficulty is to develop the analysis to a sufficient interpretative level [78]. IPA aims to understand the experiences (phenomenology) and meaning-making activities (interpretation) of the research participant [18]. The analyst attempts to make sense of the participants’ interpretation of their own experience, thus creating a double hermeneutic [79]. Nevertheless, improving the interpretation level can be facilitated by borrowing techniques from the GT [80]. Beyond the individual psychological experience described by IPA, the GT emphasizes its particular societal, cultural and political context. That is, participants’ views convey some knowledge about beliefs and practices shaped by professional norms and institutions’ organization. IPA is better known than GT in the medical field due to the historical fact that most mental health qualitative research has been designed by psychologists in order to grasp individual experiences [81]. In the present context of positive mental health, Grounded Theory provides a particularly relevant approach for qualitative researches assessing the local impact of contemporary health system changes.

The sampling strategy was to encounter diverse range of youth psychiatrists working in different settings and being either actively involved in research or not [17]. However, if psychiatrists had distinctive clinical practices and attitudes toward research, they were unanimous about their impossibility to commit in prognostications in their daily work. Thus, saturation was rapidly reached. A validity criterion of qualitative studies is to expose results of the analysis to participants. Findings were directly communicated to four participants and five oral communications were addressed to more than 70 youth psychiatrists’ who agreed with our results (oral communications in the department meetings of three hospitals Cochin, Pitié-Salpêtrière, and Avicenne (Paris Region), the annual conference of de French Society of Child and Adolescent Psychiatry (SFPEADA) in Lyon, May 2016, the annual conference of French Society for Psychiatric Information (SIP) in Bruxelles, September 2016). While it is not possible to generalize from such a small scale study, the opinion reported here may reflect the perspectives of a significant segment of French youth psychiatrists. This study is first step to provide an account of early interventions implementation. The W.H.O. argues that given their historical and conceptual diversity, the efficiency of the mental health policies and practices in different countries is difficult to compare in an evidence based perspective [82]. Using the GT in different countries would offer a qualitative comparison of young people access to mental health care and a particularly relevant insight of psychosis early management implementation in psychiatric services.

## Conclusion

This study stressed the gap between the psychosis risk research and adolescent psychiatrists daily prognosis uncertainties [83]. Psychiatrists’ reluctance toward early psychosis detection was shaped by three difficulties: first the theoretical gap between research and practice; second their impossibility to make reliable prognoses; and third, their uncertainties relating to medical judgment, adolescence and the nature of psychosis. If our participants explained their reluctance toward ARMS assessment by the clinic/research dichotomy, their inconspicuous risk management appears merely supported by the French health system facilities. They allow free follow ups that are not conditional on ARMS assessment. Improving the early management of child and adolescent mental health disorders [84] is a necessity that increases the need to acknowledge uncertainty [85]. Meanwhile, daily predictive practices appear shaped by distinctive cultural, societal and political contexts, such as mental health services design and costs coverage.

## Supporting information

S1 Appendix A supporting information file with relevant excerpts. (DOCX)
