# Supplementary material for: Psychosis risk research versus daily prognosis uncertainties: A qualitative study of French youth psychiatrists’ attitudes toward predictive practices
Source: PLoS One. 2017 Jul 19;12(7):e0179849. doi: 10.1371/journal.pone.0179849 (PMC5516970; doi:10.1371/journal.pone.0179849)
Supplement: S1 Appendix — (DOCX) [file pone.0179849.s001.docx]

« Enfin si si, c’est évident, que…. je pense que quand on reçoit un adolescent, enfin, je pense en particulier à un patient dont je m’occupe actuellement, qui est adolescent… heu les questions que je me pose quant à son pronostic, disons son diagnostic et son pronostic, viennent interférer avec la façon dont je m’en occupe. C’est à dire que je pense que ça … même si moi il se trouve que je suis son psychothérapeute, donc je suis sensée pouvoir me libérer un peu de la question… je dirais… symptomatologique, pour pouvoir permettre d’élaborer quelque chose par rapport à ce qui lui arrive. Il est évident que le fait que j’ai en tête que ce patient …. disons probablement délire … heu… même si c’est une question qui est débattue entre les gens qui s’occupent de lui, moi et d’autres personnes qui s’en occupent qui n’ont pas tout à fait cette vision. Disons que le fait que moi j’ai ça en tête vient incontestablement modifier la façon dont je m’occupe de lui. Dont je l’écoute. Je suis certainement dans une position plus thérapeutique, au sens… pour parler simplement, je me méfie de ce qui pourrait survenir […] Effectivement, moi je me pose la question : est-ce que ce patient va devenir schizophrène ? Est-ce que finalement, il va arriver à contenir ça, et qu’il restera fragile mais que bien accompagné, il va pouvoir vivre avec ça ? Moi je ne sais pas. » (psychiatre 1)

« Moi, ce qu’il faut dire c’est que je suis psychiatre, bon. Mais, au fil des années, comme je suis psychanalyste, et surtout psychanalyste, j’ai renoncé à travailler en tant que prescripteur auprès de mes patients. Pour être uniquement, soit psychothérapeute dans mon activité libérale, soit en CMPP, dans une activité de consultation, ou de psychothérapie, ou de consultation familiale. Mais en tout cas, plus dans une position de prescription. Donc quand j’ai des patients dont il m’apparait qu’ils relèvent d’une prescription, à ce moment-là je travaille en binôme avec quelqu’un. Donc j’ai des correspondants, psychiatres, qui sont prescripteurs, et avec lesquels j’ai l’habitude de collaborer. Et je leur adresse des patients, pour lesquels je reste psychothérapeute, et je leur demande de prendre en charge la prescription médicamenteuse. » (psychiatre 1)

« je ne sais pas très bien comment définir la prévention. Mais… traiter un patient, c’est-à-dire être thérapeutique, c’est quand même être dans la prévention qu’il aille plus mal ! [rires] Qu’est-ce que je pourrais dire de ça ?... Moi j’ai une idée de la prévention, là vraiment, chevillée au corps, c’est-à-dire que je pense que si on s’occupe… Parce qu’il se trouve que je suis psychanalyste pour des adultes, pour des adolescents, et pour des enfants, que si on s’occupe des gens ça va déjà mieux ». (psychiatre 1)

« (LB) Est-ce qu’il est possible de réduire un risque de transition vers la psychose, par une prise en charge?

- Alors, franchement, la survenue de la schizophrénie, là j’en suis vraiment pas sûre. Je pense qu’on peut prévenir un certain nombre de choses. Je pense que plus tôt on s’occupe des difficultés des enfants, des adolescents… Mieux on, enfin, plus on permet une évolution favorable. Dans une certaine mesure. C’est-à-dire ça dépend de quoi il s’agit ! Mais s’il s’agit de la schizophrénie, dire que, parce qu’on va s’occuper d’un enfant ou d’un adolescent, on l’empêchera d’être schizophrène… Je ne vois pas comment est-ce qu’on peut le démontrer, déjà ! Parce qu’il y en a qui ne vont pas le devenir… est-ce qu’ils le seraient devenus sans nous, qui pourra le dire ? Il y en qui vont le devenir. Est-ce que si on avait fait autrement, qu’on s’en était plus occupé, plus tôt et cetera, ils ne le seraient pas devenus ? C’est impossible de le dire aussi. Donc ma conviction, moi, je ne suis pas du tout sûre qu’on puisse empêcher un patient de devenir schizophrène. (psychiatre 1)

(LB) Justement, est-ce que vous avez connaissance des travaux des équipes qui travaillent sur les risques de psychose ? Qui font des cohortes et voient ce que les gens deviennent ?

- Oui, je vois. Mais j’ai pas lu les travaux, non. Moi je pense que c’est très compliqué de démontrer quoi que ce soit. » (psychiatre 1)

« Moi j’ai en tête que je ne sais pas du tout ce que sera son avenir, c’est-à-dire, je ne sais pas. A mon sens il est déjà délirant, même si au fond, c’est quelque chose qui n’est pas très explicite, qui n’apparait que si on approfondi quand même… Si on en reste à la surface, on pourrait penser qu’il ne l’est pas. Moi je pense qu’il l’est. … Mais je ne sais pas ce qu’il en adviendra. Effectivement, moi je me pose la question : est-ce que ce patient va devenir schizophrène ? Est-ce que finalement, il va arriver à contenir ça, et qu’il restera fragile mais que bien accompagné, il va pouvoir vivre avec ça ? Moi je ne sais pas. … Est-ce que tout ça ne sera pas qu’une espèce d’émergence, au moment de l’adolescence, dont il ne va finir par récupérer, à peu près totalement ? Avec peut-être quelque chose qui apparaitra dans plusieurs années d’ailleurs. Ou peut-être pas. Peut-être que c’est un patient qui va arriver à s’équilibrer, malgré ses difficultés psychiques, qui sont apparues de façon très manifeste au cours de l’année dernière. Donc c’est vrai que c’est un patient, c’est pour ça que je dis que je ne sais pas très bien son devenir. » (psychiatre 1)

« (LB) Vous connaissez un peu les travaux des équipes sur les risques de psychose ? Qui font des cohortes de jeunes patients avec quelques symptômes, et qui veulent voir s’ils font une transition psychotique ensuite ?

- Non. D’abord, c’est vieux comme la lune. Et puis, je ne vois pas très bien l’utilité. Ça sert à quoi ?

(LB) Il y a l’idée de la prévention… ?

- Ah oui ? [rires] Quelle prévention ? On te prévient « attention, bientôt tu vas avoir des idées bizarres » ?! Alors, dis-moi. Alors, comment on fait pour prévenir ?

(LB) [rires]. Ok. Mais l’idée c’est que si on peut prédire tôt, on pourra mieux prévenir les conséquences.

- Oui. Mais ça c’est de la clinique ! Oui, je ne pense pas que ça marchera. Et puis, après coup tout prouve tout ! «*Ah ça c’était déjà la schizophrénie …* ». Enfin, moi ça ne me sert pas à grand-chose, quand je vois un gamin de 15 ans, de me dire « *ah bah, il va être schizophrène ».* Ça ne me sert pas à grand-chose.

(LB) Oui. Mais vous y pensez quand même ou pas ?

- Oui ! oui.» (Psychiatre 2)

« Ah oui, les demandes des parents, alors… Quand il y a des demandes de prédiction, ce n’est pas tant par rapport au pronostic, que par rapport à la scolarité en général. Est-ce qu’il va réussir ? Est-ce qu’il va remonter ces notes ? Est-ce qu’il va avoir tel ou tel examen ? Dans notre fonctionnement social, l’inquiétude des parents par rapport aux enfants, en tout cas ceux qu’on reçoit, tourne beaucoup autour du scolaire. Les parents s’inquiètent, par le prisme du scolaire au début. Après, on peut éventuellement réussir à faire basculer le discours sur « comment votre enfant se construit ? Est-ce qu’il y a des inquiétudes sur son devenir ? Est-ce qu’il est en train de… d’acquérir une autonomie qui va lui permettre de fonctionner en tant que personne dans son existence ? » **(psychiatre 3)**

« On se dit que si on arrive à gagner du temps et cetera… Ça n’ira pas trop mal. Mais ce qui serait intéressant je pense, dans cette histoire d’adolescence dont on a parlé aujourd’hui, ce serait d’arriver à délimiter justement ce qui revient à une espèce de crise d’adolescence –on peut voir toutes sortes de choses d’ailleurs – et puis ce qui correspond vraiment à la psychopathologie psychiatrique. A savoir qu’au fond quand on est en train de travailler avec des adolescents, et qu’on a cette impression, d’illusion, qu’on est absolument sûr d’avoir un effet qui fait que l’adolescent ne bascule pas du mauvais côté. Est-ce que c’est vrai ou bien est-ce que c’est juste l’illusion des psys ? Est-ce qu’on leur évite… ? Peut-être pas. Et peut-être que si [rire]. C’est très vivant comme période, l’adolescence ! ... Ouais. … Ouais. … Enfin moi quand même je serais tenté de penser que si on ne fait rien à un certain moment les choses vont quand même plus mal. Vous ne pensez pas vous ? Mais ça doit être difficile à démontrer ! De toute façon, dans le doute moi je pense qu’il faut plutôt faire quelque chose que de ne rien faire. » **(psychiatre 4)**

« Je connais le centre expert autisme, mais c’est différent. Et je sais qu’à [nom d’un hôpital psychiatrique] ils sont très très… études tout ça. Mais un centre-expert, non. Alors déjà, je ne connais pas. Mais j’imagine le concept. Ça fait très peur. Non, mais si. Enfin, j’imagine qu’ils cochent « qui fume du cannabis », « qui a déjà fait un épisode », j’imagine ce genre de choses. … Bon, pour la recherche, oui, probablement que cela peut avancer des choses. Mais c’est toute la question d’anticiper une maladie qu’on n’a pas encore ! Enfin, je trouve ça assez compliqué, ça peut faire assez peur. Après, on a des traitements pour la schizophrénie, mais je, je… Moi, je ne sais pas… Enfin, je pense que je garderais en tête, s’il y a un certain nombre d’éléments peut-être qui m’inquiètent, pour une évolution vers… Où je me dis que peut-être ce jeune, il pourrait évoluer comme ça, je l’aurais en tête. Enfin voilà. Je le garderais dans un coin de ma tête, peut-être en réévaluant de temps en temps. Pas à toutes les consultations, pas tous les mois, mais de temps en temps. » **(psychiatre 12)**

« Est-ce que parler de détection ça a un sens pour leur travail ? Ou de prévention ?

Oui. Oui, c’est de la prévention secondaire. Je suis convaincu de l’intérêt de ce travail-là avec de grands adolescents, oui.

(LB) Est-ce que vous pensez que cela pourrait permettre d’éviter la survenue d’une psychose ?

- Là, je … (soupir)… Je suis convaincu qu’un certain nombre de jeunes, par leurs processus de remédiation, vont éviter des aggravations excessives en tout cas. Par rapport aux risques évolutifs de la schizophrénie sur lesquels vous me mobilisez, là, aujourd’hui, je pense que pallier à ces risques évolutifs c’est renforcer tout le réseau thérapeutique autour de l’adolescent et du jeune adulte. De le stabiliser. D’intégrer un travail de réinsertion profess… heu… enfin scolaire ou professionnelle. Enfin tout ce qu’on fait classiquement avec des personnes qui vont s‘avérer après quelques années d’évolution malgré tout dans la schizophrénie. Et pour le coup, moi je voyais en tout cas dans le passé, des formes évolutives de schizophrénies aller vers ces autismes schizophréniques qu’on nous décrivait. Que je vois beaucoup moins aujourd’hui. Mais je pense qu’un des risques évolutifs de ces personnes aujourd’hui, c’est quand même une désocialisation, un éloignement. Voire même une trop grande distance avec des systèmes thérapeutiques, comme les hôpitaux de jour ou autres. Donc cette palette dans le lien social des différentes possibilités, elle me semble mieux abordée quand on travaille par la remédiation. Mais pas uniquement ! Je pense qu’il faut aussi des psychothérapies individuelles. J’utilise beaucoup le psychodrame, avec des jeunes schizophrènes, psychotiques. Et je vois que cela contribue vraiment à de bonnes stabilisations. A l’apparition quand même d’une autonomie possible. Dans l’hébergement.

(LB) C’est possible d’obtenir une autonomie normale ? Vous y arrivez ? Enfin je dis « normale », mais voilà, comme quelqu’un qui n’aucun problème, tout simplement.

- Alors, moi je dirais… Pour moi, les personnes que je vois redevenir « normales » pour prendre votre mot, je suis obligé de me dire, quelques années après, que je m’étais trompé sur le diagnostic de schizophrénie. Vous voyez ? Et que je l’avais même mis en place trop tôt dans ma tête. Je vais vous donner un exemple. » **(psychiatre 5)**

« LB - Est-ce que vous connaissez un peu les travaux sur les risques de psychose chez les adolescents ?

- Les centres experts vous voulez dire ? Oui oui. Sur la schizophrénie je ne les ai pas encore utilisés… Heu… Je n’ai pas un a priori défavorable. Quand ils sont venus se présenter ici, par exemple le centre expert de ////, je n’ai pas … heu… Je me suis dit que je m’en servirais oui, pour certains patients. Mais, je trouve que la batterie de questions si vous voulez – autant sur la bipolarité ça me semble très clair qu’il faut aller très très tôt vers ces batteries-là pour clarifier un projet thérapeutique – autant sur la schizophrénie je dirais, c’est peut-être prétentieux, mais j’ai tendance encore à me faire confiance pour me laisser évoluer. Y compris avec le flou des premiers mois, parfois plus. Plutôt que d’exposer le patient et sa famille à une batterie de questions qui diront « vous êtes psychotique ou schizophrène ». Alors, en même temps je suis méchant par rapport à ce que l’on nous a dit, parce que les confrères qui sont dans ces centres experts disent bien, eux aussi, qu’il faut attendre cinq ans. Ils disent bien eux aussi qu’il faut être très prudent, qu’on n’a pas une sécurité à 100%. Voilà. Mais, cette interface quand même, de la batterie de travail qu’ils vont mettre en place, à la limite je dirais que je préfère utiliser ce type de travail pour des professionnels qui ne sont peut-être pas dans la psychiatrie. Qui vont avoir ces jeunes-là chez eux…

LB - Par exemple ?

- Des services éducatifs par exemple. Ou des services de protection. Et je leur dirais « mais il faudrait que vous voyez un médecin qui vous aide à orienter ce garçon vers un centre expert pour que l’on comprenne si ce garçon est effectivement dans ce type d’évolution ». Vous voyez ? Donc pour le coup, là je pense que de clarifier la situation aiderait l’environnement éducatif - soit à se rassurer sur le fait que c’est strictement éducatif – soit à se rassurer sur le fait qu’ils ne peuvent pas tout faire et qu’il va falloir vraiment mettre en place de la psychiatrie pour aider ce garçon.

LB - Donc, si je comprends bien, vous dites que le centre expert c’est intéressant, mais que cela peut-être trop précoce ?

- Dans le travail que moi je fais avec mes patients, oui, je le crains.

LB - Qu’il vaudrait mieux que ce soient d’autres acteurs sociaux qui utilisent le centre expert ?

- Pas pour le même patient par contre. Si moi je travaille, avec ce patient-là, cette hypothèse du diagnostic je préfère la garder pour moi. Mais quand des équipes éducatives viennent me présenter ici à la maison de l’adolescent, un problème qui me fait penser que le risque psychotique est là, quasiment, voire déjà évolutif… A ceux-là, qui n’ont pas de psychiatre dans leur réseau, je leur dis « il faudrait que vous rencontriez un psychiatre qui accepte de travailler avec un centre expert sur un éclairage de ce garçon ». Vous voyez ce que je veux dire ? Parce qu’ils sont trop eu outillés, et ils n’ont pas… Enfin, voilà. Et que cela désorganise beaucoup leurs actions éducatives que des troubles sur les compétences relationnelles, sur les troubles cognitifs, ou de dysharmonie, viennent perturber des choses qu’ils mettent en place. Par exemple des insertions chez un artisan… dans une famille d’accueil… et que cela casse à chaque fois.

LB - Donc si je comprends bien, pour vous en tant que psychiatre, le centre expert vient faire doublon à votre travail ?

- Pour les psychotiques hein. Pas pour les bipolaires, on est bien d’accord. Pour les bipolaires j’aime bien l’aide du centre expert, parce que parfois il va plus vite que moi sur un diagnostic, et là l’urgence du médicament me semble… aller de pair si vous voulez. Alors qu’avec des troubles psychotiques, l’urgence du médicament me semble être associée au regard du prescripteur, vous voyez ? Et comme les centres experts ne veulent pas devenir des centres de suivi... Ils ont raison. Voilà. Mais bon. C’est adossé à une absence d’expérience sur le centre expert schizophrène.

LB - C’est nouveau oui…

- Alors que sur le centre expert bipolaire, j’y vais plus volontiers.

LB- Donc le centre expert aurait plus vocation à être tourné vers la population générale ?

- Ah je crois oui. Je crois. De mon point de vue hein. Maintenant, pour de jeunes professionnels comme vous, peut-être que… Ça fait partie aussi de l’organisation du réseau de soins autour d’un schizophrène, que de s’y engager avec un centre expert. Donc, sur le fond, je trouve important que ces centres existent. Voilà. Après c’est comment on les utilise, avec quelle précision, dans le projet… Enfin.

LB - D’accord. Et bien, merci beaucoup, c’était très intéressant.

- Merci à vous. » **(psychiatre 5)**

« Mais par rapport au premier cas, donc, ce patient je l’ai suivi longtemps, 4 ans, jusqu’à sa majorité. Là, le relais en psychiatrie adulte s’est fait là, il y a trois semaines. C’était, finalement, là, même moi là, je ne suis pas sûre du diagnostic ! … Mais à la fois, je trouve que dans l’évolution actuelle de dire « il faut qu’on donne un diagnostic absolument », les parents *viennent* avec cette demande-là… Des bilans *en vue* d’un diagnostic et cetera. Alors on bilante et on fait des diagnostics multiples [rire] qui sont une sorte de listing de diagnostics ! Mais bon, au même temps on les a faits. On a bilanté et cetera. Mais là finalement je… je ne me suis jamais prononcée clairement sur un diagnostic -qui serait effectivement un diagnostic de trouble chronique - ce qui n’a pas été forcément simple pour moi dans la justification de la poursuite d’un traitement, chez le jeune. Parce que ça, il prenait un traitement retard, et il demandait – surtout à la fin – pourquoi il le prenait. Et il disait que je n’avais jamais bien réussi à le lui expliquer… [rire] » **(psychiatre 7)**

« C’est une des questions sur le risque de psychose…Entre schizophrénie et trouble de l’humeur, hein. Parce qu’il y a des situations cliniques où les premiers épisodes sont très très très bruyants, très psychotiques, très délirants et cetera… Mais qu’il y a quelque chose, cliniquement, qui te fais dire que c’est plutôt un trouble bipolaire. … Mais bon. Au même temps, on a fait une étude de devenir, à laquelle j’ai participé. Sur 80 adolescents âgés entre 12 et 19 ans au moment de leur hospitalisation, avec tous un diagnostic de trouble bipolaire type I. … Et, *un tiers*, à l’âge adulte - donc à 8 ans en moyenne de devenir – sont dans un spectre schizophrénique !

(LB) Donc la question du devenir elle se pose aussi, là.

- Oui. Oui. Alors, bon, on pouvait aussi répondre, en tout cas méthodologiquement, mais « est-ce que vous étiez *bien* sûrs que c’était un état maniaque ? Ou est-ce que c’était une BDA ? ». Rétrospectivement bon. Mais enfin, tous les patients avaient été connus par un médecin du service. Mais quand même : *un tiers* !!! A dix ans de devenir. Donc jeunes adultes et tout ça. Pour lesquels il y avait un diagnostic de trouble schizoaff’ ou schizophrénie ! Donc double question. Là, je me fais moi-même ma discussion dialectique. Parce que cette jeune… ! J’en ai connu aussi une autre… heu… un tableau hyper hyper floride, mais elle avec une note maniaque claire, mais très délirante et tout… Enfin, et … et très désorganisée et cetera. J’étais *sûre* qu’elle était bipolaire ! Et pour la patiente dont tu parles… *aussi*! Donc bon. Alors, c’est aussi la durée du suivi hein. » **(psychiatre 7)**

« LB : Le but est de comprendre comment tu travailles, quand tu rencontres des adolescents –soit en ambulatoire soit en hospitalisation - pour des problèmes un peu flous, et pour lesquels tu te poses la question d’une éventuelle transition psychotique. Donc ce qui m’intéresse spécifiquement c’est comment tu réfléchis dans ces situations-là, comment tu penses à l’avenir… Qu’est ce qui te pose problème.

- Alors, j’ai plein de façons de te répondre, donc c’est compliqué. Peut-être une première chose c’est de dire qu’il y a une position de psychiatre de l’adolescent, qui est de se confronter à l’adolescence. A l’immédiateté de l’adolescence. En particulier en hospitalisation, peut-être d’avantage qu’en consultation. C’est vrai que moi je travaille en hospitalisation, donc j’ai cette particularité-là d’être beaucoup dans l’immédiateté et pas forcément dans le pronostic à plusieurs années, si tu veux. Et ça, je pense que c’est une réalité quand on prend en charge les adolescents. C’est marrant, on en discutait ce matin avec mon interne. Du fait que, elle, elle me disait « j’ai du mal à comprendre certaines fois les ados ». Et finalement elle me dit « j’ai pas assez de bagage, il faudrait que je lise plus. Mais notre formation ne nous permet pas de lire suffisamment. On n’a pas le temps de construire trop sur le plan théorique ». Ce à quoi je lui répondais « je pense que chez l’ado de toute façon, c’est compliqué de le penser. Parce que dès que tu le penses, hop ! – dès que t’arrives à le penser – hop ! il a changé ». Un truc comme ça quoi. Que tu es obligé de te confronter à l’immédiateté. Et que si tu penses trop loin, le temps que tu penses, et bien il a déjà changé. Et ta pensée n’est déjà plus d’actualité. Donc ça c’est une première chose qui fait qu’en termes de pronostic, on n’est pas dans des … … et je pense que c’est pour ça qu’on a une position différente des psychiatres d’adultes. C’est que c’est compliqué de penser trop loin, si tu veux. Le risque à court terme c’est le quotidien de la psychiatrie de l’adolescent. On est dans l’immédiateté encore une fois.

(LB) Et …donc le risque à long terme il est moins présent ? Ou il n’a pas sa place ?

- Je pense qu’il est plus présent dans la tête des psychiatres que dans la tête des ados, ça c’est évident. Et que dans la tête des parents.

(LB) Et comment il est présent dans la tête des psychiatres alors ?! Moi, c’est ça qui m’intéresse !...

- [rire]

(LB) …même s’il n’est pas dit aux ados ni aux parents. Comment est-ce qu’il est présent ?

- Je pense qu’il est présent quand on s‘arrête et qu’on y réfléchit, si tu veux [rire]. Et quand on en parle entre nous [rire]. … Mais quand on est dans le travail quotidien on n’y pense pas. On n’y pense pas. … Ça arrive qu’on y pense. En termes de, effectivement, quand on se dit « *Cet ado, il est tellement fragile. Comment ça va se… comment ça va se terminer ?*». On ne sait pas. On ne sait pas. …Donc… bah, on reste avec nos questions, on n’a pas de réponse quoi.» **(psychiatre 8)**

« LB - Est-ce que c’est possible d’éviter la survenue d’une psychose?

Ha ! [rire] Bonne question ! [rire]. Je serais curieux d’avoir la tendance de tes réponses auprès des psychiatres ! … (sérieux) Je ne sais pas. Je ne sais pas. Je ne sais pas. Je vais te répondre de manière, enfin peut-être à côté. L’interne qui me parlait ce matin, elle disait « c’est compliqué les ados, des fois je me sens nulle, et je ne sais pas comment faire ». Alors, elle rapporte tout de suite ça à la théorie en disant « Je n’ai pas assez de bagage théorique ». Je lui dis : mais non ! Il y a un certain nombre d’ados pour lesquels on ne sait pas comment on fait, on ne sait pas ce qu’on fait, on a l’impression d’être nuls, et les ados vont mieux. … Et c’est… finalement, c’est souvent quand on se sent nul que les ados vont mieux. Et je lui disais c’est peut-être aussi, à mettre en parallèle avec la façon dont on peut comprendre l’adolescence. Ce matin, dans les consultations de groupe, on se demandait « quand est-ce qu’on devient adulte ? Et qu’est-ce que ça veut dire d’être adulte ? ». Finalement, il y avait une des psychiatres qui disait « finalement quand on est adolescent, on perd toutes les illusions de toute-puissance des parents, et de parents super-héros, et qu’on se rend compte que nos parents sont nuls. Et devenir adulte, c’est le moment où on devient indulgent avec les failles et les faiblesses de nos parents et que l’on fait avec ce qu’ils peuvent nous apporter ». Alors, c’est parfois quand on est nuls que ça marche le mieux. Parce qu’on devient une figure d’identification possible. Quand on est trop fort finalement, pfff, les ados, ils ne peuvent pas s’accrocher à rien du tout de nous. Et on ne les aide pas quoi.

Donc… l’idée c’est de…. ?

On fait ce qu’on peut. Je pense qu’il faut faire ce qu’on peut. Ce qui est important ce n’est pas de vouloir tout le temps bien faire. C’est de faire au mieux, de savoir qu’on fait parfois des erreurs. Que ça arrive. De pouvoir les assumer aussi. Je pense que c’est important. Et, de servir aussi à certains moments de figure d’identification. Je pense qu’on a ce rôle-là, qui n’est pas forcément vrai pour l’adulte. Peut-être un peu dans la psychose. Dans la psychiatrie adulte, les psychiatres adultes qui s’occupent des pathologies limites graves, des psychoses, ils sont peut-être dans une proximité comme ça avec les patients, un peu comme la nôtre. Je pense que les adolescents, ils ont des fonctionnements psychotiques normaux à certains moments [rire]. Dans leur façon d’être en lien. C’est normal. Ils sont entre deux, hein. Ils sont en train de se structurer. Ils ont des mécanismes d’enfant, de projection, et de choses qui peuvent se rapporter à la psychose. Et puis d’autres mécanismes d’avantages névrotiques. Donc voilà ! Je n’ai pas de réponse à te donner. Je ne sais pas, je ne sais pas. J’aimerais bien que ça serve à quelque chose, et je pense que ça sert à quelque chose. Si j’étais persuadé que ça ne servait à rien, je pense que je ferais autre chose. » (8)

« on ne fait pas de la météo, quoi ! [rire] Tu vois le terme de risque… Ça reste des probabilités quoi ! Ça reste des statistiques ! Si on me donne la recette magique qui me fait dire « celui-là, il va être psychotique », je veux bien la prendre [sourire]. Ca règlerait pas mal de questions hein, mais au même temps ça rendrait le boulot un peu chiant, moi je pense [rire]. Je pense que c’est ça qui est intéressant aussi, c’est de ne pas savoir, dans ce qu’on fait. … Hmm… (soupir). C’est compliqué, hein. Et je pense qu’il faut en tenir compte bien sûr. Et je te caricature un peu le… Mais … … Tu vois. Moi je pense en termes de risque à court terme, encore une fois, avec les ados. Et je ne pense pas en termes de risque à long terme. Je ne pense pas les ados dans 20 ans, ou dans 10 ans. » (8)

« (LB) Donc, ma première question serait de savoir si le pronostic en général est un enjeu dans votre pratique.

- (soupir) C’est un enjeu dans quel sens ?

(LB) Est-ce que c’est une question qui se pose dans votre pratique ? Qui selon vous fait partie du travail du psychiatre d’adolescents ? De faire des pronostics pour soi déjà et peut être aussi après pour en parler…

- Ah bah c’est une question difficile à laquelle répondre… parce que… (Soupir) Comment dire ? C’est tellement variable à l’adolescence. On se base un peu parfois sur les statistiques, ou sur des statistiques personnelles quand on arrive à identifier certains profils. Parce que je pense que pronostic demande diagnostic. Et… on est souvent embêté avec la question du diagnostic je dirais à l’adolescence. Le fait que ce ne soit pas… L’adolescence, si on suit les nouvelles études qui sont en train de nous montrer qu’elle se termine vers vingt-cinq ans, donc pas à l’âge adulte… Heu, je dirais que les diagnostics, oui, on peut mettre des diagnostics symptomatiques. A part quand on est vraiment convaincu que c’est une rentrée dans la psychose donc quelque chose qui risque d’aller dans la structuration de la personnalité. Mais on est confronté tellement à une plasticité encore - l’avantage de l’adolescence c’est qu’il y a tout un tas de modifications possibles - qu’on est plus dans un fonctionnement que dans une structure. Dans un fonctionnement de la personne, que dans la structure de la personnalité. Donc moi je dirais que le pronostic, vu que le diagnostic on essaye justement de ne pas les donner, cela risque de créer encore plus en psychiatrie, je dirais une étiquette, quoi. ‘’Je suis ça’’. Et peut être encore plus pour les parents que pour le patient. Donc je dirais que le diagnostic on le nomme, à part les symptômes dépression et tout cela anorexie …

(LB) Effectivement moi le cas qui me pose question c’est celui de l’éventualité d’une évolution vers la psychose.

- Oui c’est plutôt ça. Heu, après cela dépend de tellement de variables, parce qu’il n’y a pas un schizophrène qui est comme l’autre et heureusement quoi ! Après, toutes les composantes déjà de l’atteinte de la personne, de son âge, des solutions qu’on arrive à mettre en place, et de tout l’étayage environnemental. Familial et tout le reste

(LB) Et pour la partie du pronostic est ce que c’est quelque chose qui vous pose question, mais avec lequel vous êtes un peu embêté pour travailler ou alors est ce que c’est quelque chose qui ne se pose pas comme question ?

- Non, non ! Alors la question se pose toujours, et je suis bien embêté (rire gêné). …Heu, alors. Si on est dans le cadre de la psychose, je dirais déjà qu’on essaye de voir comment le jeune a pu évoluer au cours de l’hospitalisation ici et de voir quels sont les moyens qui sont mis en place à l’extérieur. » **(psychiatre 10)**

(Psychiatre 10) Mais là c’est une polémique de ma part, je trouve qu’on emploie en France avec trop de facilité le mot schizophrénie, moi je dirais que c’est plutôt ça, qu’on emploie trop facilement le mot schizophrénie. Entre nous, pas tellement avec les familles

(LB) Ah oui, dans le langage courant ?

Mais même entre médecins la plupart des fois et même si vous rencontrez des soins-études, combien de fois les patients ont été étiquetés comme schizophrène et avec le temps ils voient qu’ils ne l’étaient pas. Combien de fois ils changent le diagnostic parce que la personne avec un étayage, avec des soins… Ou ils n’étaient pas schizophrènes, ou ils ont réussi à se soigner, à circonscrire…

(LB) Oui c’est exactement ça ma question : comment est-ce que on fait quand il y a cette possibilité mais qui n’est pas une certitude, où il y a cette idée que cela pourrait peut-être évoluer vers une psychose ?

Eh bien c’est là le tort. Et c’est là où je pense qu’il faut faire attention de nommer à ce moment-là. Si on a un doute, à mon avis il vaut mieux ne pas le nommer. Il vaut mieux éventuellement parler de psychose qui fait un peu moins peur… heu… et surtout inciter le travail sur la suite, et bien voilà ! … C’est vraiment le temps futur qui peut dire ça.

(LB) Est-ce que la prise en charge vous avez l’impression qu’elle peut éviter, justement, ben la survenue d’une schizophrénie si ?, Est ce que parfois vous avez l’impression que cela peut être évité ?

…heu… (long silence, soupir) … Alors … (soupir). Vous me posez des questions difficiles vous ! (rire)

(LB) Ouais c’est dur

… heu je n’en sais rien. Dans le sens que on peut miser sur le fait que si ils n’ont pas de soins adaptés, appropriés, je ne sais s‘ils vont devenir vraiment schizophrènes, mais c’est sûr qu’ils risquent encore plus de s’appauvrir. Voilà. Psychiquement, cognitivement. Ne pas avoir de soins dans des situations qui sont à la frontière, ça risque de repousser l’accès aux soins et que la plupart des fois c’est à travers d’un passage à l’acte et de quelque chose d’explosif que la famille qui a réussi à tenir autour d’un équilibre précaire, va certainement ne va plus avoir le choix, et va l’adresser sur le secteur. Après cela c’est au secteur de nous les adresser à nous, pour qu’on travaille toute la suite. Sans soins, je ne saurais jamais. Après il faudrait prendre le risque d’avoir une cohorte de patients qu’on prend en charge, d’avoir l’équivalent qu’on ne prend pas en charge, et on suit l’histoire naturelle. Déontologiquement, ça c’est compliqué. … Je ne sais pas si je réponds pertinemment à vos questions ?

(LB) Oui, moi mes questions portent sur les opinions, sur comment vous voyez les choses.

Moi je pense qu’il y a plus, si le patient est réellement en train de rentrer dans la schizophrénie, il y a plus de risques que sans soins, qu’il s’y enfonce. Qu’il y ait vraiment un appauvrissement. **(Psychiatre 10)**

«  Pour moi le pronostic c’est… Il y a plusieurs choses. Il y a le pronostic au niveau d‘une maladie ; on parle des adultes-là ? Ou des jeunes adultes ? Parce que je ne vois pas forcément ça de la même manière chez des enfants, chez les ados, et chez les adultes. **(psychiatre 12)**

Les chercheurs s’intéressent par exemple à ce qu’on voit dans les troubles des apprentissages et qui pourrait être prédictif d’une transition psychotique. Ça c’est leur hypothèse. Bien sûr. Ça c’est leur hypothèse. Et je pense qu’on peut voir des choses ! Mais c’est toujours la même chose : mais qu’est-ce qu’on va en faire ? Qu’est-ce qu’on va en faire ? Moi, je ne dirai jamais… heu… Parce que, je vois bien les profils de patients qui les intéressent et les profils de patients pour lesquels, moi je me pose des questions. C’est typiquement les gamins, qu’on appelle dyspraxiques. Qui ont un rapport à l’objet, pas au point. Dès qu’on les met à manipuler, et à faire tout ce qui est spatial etc, ça ne va pas. C’est depuis la petite enfance. Tant que la maman est là, à faire les chaussettes et cetera, l’habillage, tout se passe bien. Quand… après, ça va moins bien. Quand, au moment du raisonnement logico-maths on passe à l’abstraction, ça ne va pas ! Bon. C’est tous ces petits signes-là. Mais moi je ne me vois pas dire que j’ai observé ça, et dire à des parents « bah voilà, votre fils-là, il vient de rentrer au collège. L’abstraction, ça ne va pas du tout ». Enfin, ça je peux le dire. Mais « par contre dans les équipes de recherche, on pense qu’il y a, qu’il y a, qu’il y a 30% de risques qu’il y ait une émergence psychotique à l’adolescence !! ». Bah ouais, mais ça c’est pas possible !! On ne peut pas faire des ch[oses comme ça]. On peut peut-être le penser, on peut faire un travail de recherche là-dessus, mais *en clinique*, ce n’est pas possible ! Et *par contre*, que ces gamins-là, moi j’ai l’œil dessus, parce que je sais que… de temps en temps ça vire pas bien, ça oui. Mais, mais je conseille que le suivi soit continué, je donne des adresses, quand ils arrêtent ici je dis qu’il faut continuer etc. Oui ! Mais, le, le fait que des chercheurs soient sur la transition psychotique, bah, c’est des questions de chercheurs ! Ça n’a pas à être dit en clinique. **(Psychiatre 6)**
